# Supplementary material for: Shared and distinct roles of Esc2 and Mms21 in suppressing genome rearrangements and regulating intracellular sumoylation
Source: PLoS One. 2021 Feb 18;16(2):e0247132. doi: 10.1371/journal.pone.0247132 (PMC7891725; doi:10.1371/journal.pone.0247132)
Supplement: S1 Table — At least two independent isolates per mutant were used. The results are plotted in Figs 1C, 1D, 2F and 7C. (DOCX) [file pone.0247132.s001.docx]

**S1 Table.** Rates of accumulating dGCRs are measured using fluctuation analysis. At least two independent isolates per mutant were used. The results are plotted in Figs 1C, 1D, 2F and 7C.

| **Genotype** | **dGCR Rate** | **Ratio** |
| --- | --- | --- |
| Wild type* | 1.97x10^-8^ [4.32x10^-8^ – 1.16x10^-8^] | 1 |
| *esc2∆** | 2.40x10^-6^ [3.62x10^-6^ – 1.79x10^-6^] | 122 |
| *mms21-CH** | 4.20x10^-6^ [2.22x10^-5^ – 2.40x10^-6^] | 213 |
| *esc2∆ mms21-CH* | 2.21x10^-5^ [3.25x10^-5^ – 1.11x10^-5^] | 1120 |
| *rrm3∆** | 3.87x10^-8^ | 2 |
| *rrm3∆ mms21-CH*** | 9.74x10^-8^ [5.77x10^-8^ – 2.28x10^-7^] | 5 |
| *rrm3∆ esc2∆* | 1.24x10^-5^ [2.07x10^-5^ – 2.22x10^-6^] | 630 |
| *pol32∆** | 3.15x10^-8^ | 2 |
| *pol32∆ mms21-CH*** | 2.75x10^-7^ [6.82x10^-7^ – 1.11x10^-7^] | 14 |
| *pol32∆ esc2∆* | 6.35x10^-7^ [1.21x10^-6^ – 1.92x10^-7^] | 32 |
| *rad9∆** | 3.82x10^-8^ | 2 |
| *rad9∆ mms21-CH*** | 1.74x10^-7^ [2.86x10^-7^ – 1.40x10^-7^] | 9 |
| *rad9∆ esc2∆* | 1.85x10^-7^ [4.44x10^-7^ – 9.02x10^-8^] | 9 |
| *rad52∆** | 1.09x10^-8^ | 1 |
| *rad52∆ mms21-CH*** | 1.20x10^-7^ [3.84x10^-7^ – 4.98x10^-8^] | 6 |
| *rad52∆ esc2∆* | 2.40x10^-7^ [7.36x10^-7^ – 6.17x10^-8^] | 12 |
| *esc2-2FA* | 2.95x10^-6^ [2.26x10^-6^ – 4.10x10^-6^] | 150 |
| *esc2-2FA (pRS423)* | 5.44x10^-7^ [9.21x10^-7^ – 3.13x10^-7^] | 28 |
| *esc2-D430R* | 1.97 x10^-7^ [3.17 x10^-7^ – 9.23 x10^-8^] | 10 |
| *esc2-D430R siz1∆ siz2∆* | 7.49 x10^-6^ [1.27 x10^-5^ – 5.83 x10^-7^] | 380 |
| *esc2-D430R mms21-CH* | 8.39 x10^-6^ [1.33 x10^-5^ – 5.36 x10^-6^] | 426 |

* Rates taken from Putnam *et al.* (2009) and/or Albuquerque *et al.* (2013)

** Rates taken from Liang *et al.* (2018).

Ratio shows the relative fold-increase in dGCR rate of cells of the indicated mutant over the dGCR rate of wild type cells.

Square brackets indicate the upper and lower bounds of the 95% confidence interval.
