# Supplementary material for: Shared and distinct roles of Esc2 and Mms21 in suppressing genome rearrangements and regulating intracellular sumoylation
Source: PLoS One. 2021 Feb 18;16(2):e0247132. doi: 10.1371/journal.pone.0247132 (PMC7891725; doi:10.1371/journal.pone.0247132)
Supplement: S5 Table — (DOCX) [file pone.0247132.s005.docx]

**S5 Table.** Yeast strains used in this study.

| **Strains** | **Genotype** | **Source** |
| --- | --- | --- |
| HZY1414 | aka RDKY6678, *can1::hisG, yel072w::CAN1/URA iYEL072::HYG,* the parental dGCR strain | Putnam *et al.* 2009 |
| HZY1442 | *esc2∆::HIS3* in HZY1414 (RDKY6678), dGCR strain | Liang *et al.* 2018 |
| HZY2425 | *mms21-CH::G418,* MAT alpha, dGCR strain | Liang *et al.* 2018 |
| HZY2447 | *mms21-CH::G418, esc2∆::NAT,* MAT alpha, dGCR strain | This study |
| HZY1427 | *esc2∆::G418, siz1∆::HYG, siz2D::NAT,* pRS316-Esc2 (HZE2014), MAT alpha, W303 background | This study |
| HZY4304 | *esc2∆::NAT, rrm3∆::G418,* MAT a, dGCR strain | This study |
| HZY4312 | *esc2∆::NAT, rrm3∆::G418,* MAT alpha, dGCR strain | This study |
| HZY4323 | *esc2∆::NAT, pol32∆::G418,* MAT a, dGCR strain | This study |
| HZY4324 | *esc2∆::NAT, pol32∆::G418,* Mat alpha, dGCR strain | This study |
| HZY3443 | *esc2∆::G418, rad9∆::NAT,* MAT a, dGCR strain | This study |
| HZY3444 | *esc2∆::G418, rad9∆::NAT,* MAT alpha, dGCR strain | This study |
| HZY4302 | *esc2∆::NAT, rad52∆::G418,* MAT a | This study |
| HZY4303 | *esc2∆::NAT, rad52∆::G418,* MAT alpha | This study |
| HZY443 | Esc2-TAF:G418, dGCR strain | This study |
| HZY479 | esc2-(2FA)-TAF:HIS, MAT a, dGCR stain | This study |
| HZY207 | *esc2-2FA(F30A, F31A)::HIS*, MAT alpha, dGCR strain | This study |
| HZY3488 | Esc2-TAF::HIS, dGCR strain | This study |
| HZY3489 | *esc2-D430R-TAF::HIS*, dGCR strain | This study |
| HZY4345 | Diploid (*siz1∆::HYG/SIZ1*, *siz2∆::NAT*/*SIZ2*, *esc2-D430R::HIS*/*ESC2*), W303 | This study |
| HZY4336 | *esc2-D430R::HIS*, MAT a, derived from HZY4345, W303 | This study |
| HZY4339 | *siz1∆::HYG, siz2∆::NAT, esc2-D430R::HIS*, MAT alpha, derived from HZY4345, W303 | This study |
| HZY4340 | *siz1∆::HYG, siz2∆::NAT,* MAT a, derived from HZY4345, W303 | This study |
| HZY2101 | Wild type, HF-SUMO strain | Albuquerque *et al.* 2013 |
| HZY2109 | *siz1∆::HIS, siz2∆::URA,* HF-SUMO strain | Albuquerque *et al.* 2013 |
| HZY3530 | *esc2-D430R::HIS*, HF-SUMO, MAT a, derived from HZY2101 | This study |
| HZY4320 | *esc2-D430R::HIS siz1∆::G418, siz2∆::URA,* HF-SUMO strain, MAT a, derived from HZY2109 | This study |
| HZY1843 | *esc2-D430R,* MAT a, dGCR strain | This study |
| HZY1846 | *esc2-D430R mms21-CH:G418*, MAT alpha, dGCR strain | This study |
| HZY1844 | *esc2-D430R::HIS siz1∆::TRP siz2∆::NAT*, MAT alpha, dGCR strain | This study |
