# Supplementary material for: Shared and distinct roles of Esc2 and Mms21 in suppressing genome rearrangements and regulating intracellular sumoylation
Source: PLoS One. 2021 Feb 18;16(2):e0247132. doi: 10.1371/journal.pone.0247132 (PMC7891725; doi:10.1371/journal.pone.0247132)
Supplement: S6 Table — (DOCX) [file pone.0247132.s006.docx]

**S6 Table.** Plasmids used in this study. Details of plasmid construction are available upon request.

| **Plasmids** | **Genotype** | **Reference** |
| --- | --- | --- |
| HZE1064 | pRS313::HIS | This study |
| HZE2779 | pRS313-ESC2::HIS3 | This study |
| HZE2014 | pRS316-ESC2::URA3 | This study |
| HZE2790 | pRS313*-esc2-2FA(F30A/F31A)::HIS* | This study |
| HZE2907 | pRS315-*esc2-2FA(F30A/F31A)-TAF::G418* | This study |
| HZE2911 | pRS423-*esc2-2FA(F30A/F31A)-TAF::G418* | This study |
| HZE2912 | pRS313-ESC2-TAF::G418 | This study |
| HZE1864 | pET21-ProteinA-6xHIS-Esc2 | This study |
| HZE2925 | pET21-ProteinA-6xHIS-esc2-D430R | This study |
| HZE2824 | pRS313-*esc2-D430R::*HIS3 | This study |
