# Supplementary figures and images for: Shared and distinct roles of Esc2 and Mms21 in suppressing genome rearrangements and regulating intracellular sumoylation
Source: PLoS One. 2021 Feb 18;16(2):e0247132. doi: 10.1371/journal.pone.0247132 (PMC7891725; doi:10.1371/journal.pone.0247132)

The following are used to generate Fig. 2B

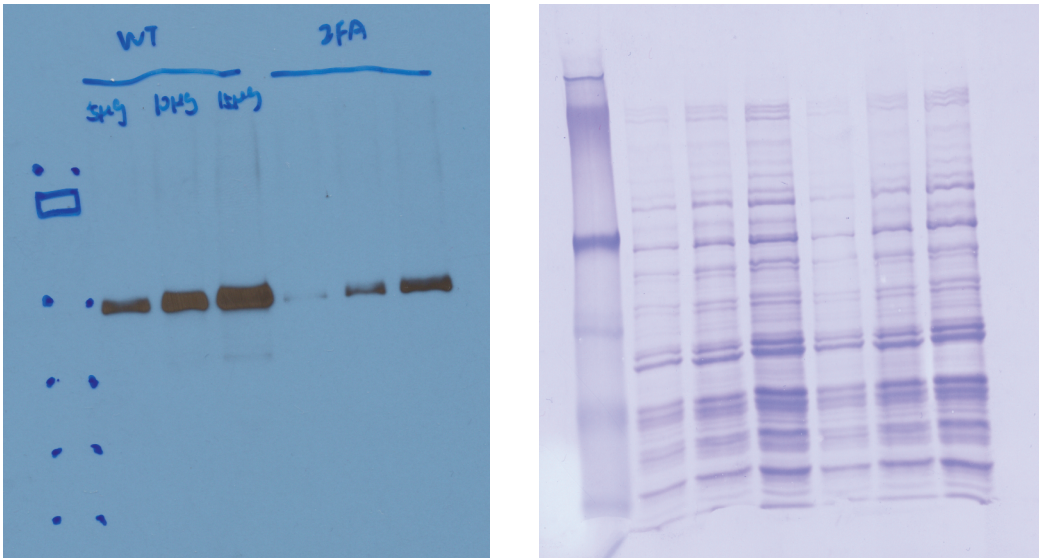

The following are used to generate Fig. 2E

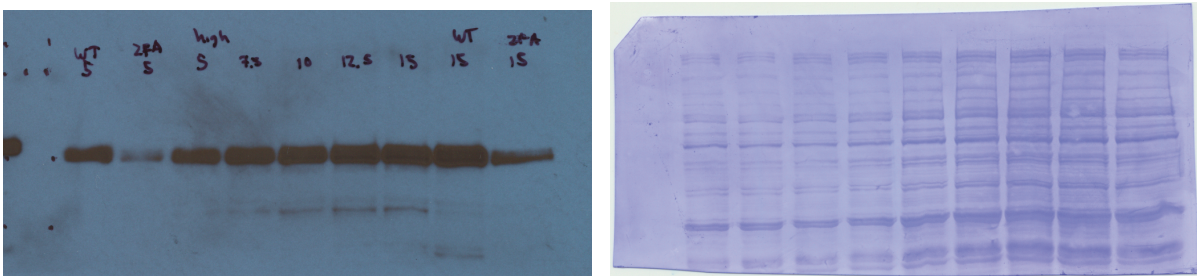

Supplement: S1 Fig — (PDF) [file pone.0247132.s007.pdf]
