# Supplementary material for: Shared and distinct roles of Esc2 and Mms21 in suppressing genome rearrangements and regulating intracellular sumoylation
Source: PLoS One. 2021 Feb 18;16(2):e0247132. doi: 10.1371/journal.pone.0247132 (PMC7891725; doi:10.1371/journal.pone.0247132)
Supplement: Attachment — Submitted filename: Respose to reviewers.doc [file pone.0247132.s008.doc]

# DEPARTMENT OF CELLULAR AND MOLECULAR MEDICINE EMAIL: [**huzhou@ucsd.edu**](mailto:huzhou@ucsd.edu)

HTTP://CMM.UCSD.EDU

January 20, 2021

Anja-Katrin Bielinsky
Academic Editor
PLOS ONE

Dear Dr. Bielinsky,

I am writing to submit the revised manuscript [PONE-D-20-36830] for consideration of publication in PLOS ONE.

We have made substantial changes to the manuscript to address the concerns from each reviewer. They include new results in the revised Figures 4 and 7, and a detailed discussion about our findings and their implications. The responses to the reviewers are included below.

The original gel/blot pictures used to generate the cropped images in the main Figures 2B and 2E are provided as Supplementary Figure 1. The gels/blots shown in Figures 3B and 3C was not altered from their original images.

Thank you very much for your effort in guiding the review of our work and we look forward to hearing from you soon.

With regards,

Huilin Zhou, Ph.D.

**Overall Responses to the Reviewers:**

First, we would like to thank both reviewers for their helpful and constructive comments. We have made substantial revisions to the manuscript to address them. Major changes include a new SUMO proteomics experiment to directly compare sumoylation levels in the *esc2-D430R* and *mms21-CH* mutants (see New Figure 7). This new result shows that these mutants have similar effects on intracellular sumoylation, supporting the model that the Esc2-Ubc9 pathway functions in the same pathway as Mms21. Additionally, we renamed the old Figure 6 to the new Figure 4, which now includes a new result about the DNA damage sensitivity of various *esc2* and *mms21* mutants as requested by the reviewers. Specific responses to each reviewer’s comments are discussed below.

**Responses to Reviewer 1 comments:**

1&2) The conserved FF motif of Esc2 is important for its protein stability in the cell, and the GCR phenotype of *esc2-2FA* points to other functions that cannot be explained by protein abundance alone. We do not know whether this FF motif of Esc2 mediates protein-protein interactions, since both our candidate testing and unbiased pull-down MS experiments have not revealed any promising candidates. We also cannot exclude the possibility that this FF motif of Esc2 may play other roles that do not involve protein-protein interaction. However, two reasons promoted us to report this finding. First, we feel it is always important to report novel findings that can stimulate further studies by the research community. Second, this result does not adversely affect the main conclusion of this study.

3) To date, the function of MCM sumoylation in preventing GCRs is unknown. The findings that Mms21 and Esc2 regulate MCM sumoylation suggest that future study to investigate this topic is potentially interesting, but it is beyond the scope of this study. Because of this, we have removed the discussion about this in the introduction section to avoid a misleading impression about the focus of our study.

4) See new Figure 7 for a direct comparison of sumoylation levels in the *esc2-D430R* and *mms21-CH* mutants. This new result shows that the levels of sumoylated proteins are similar with relatively modest differences (<2 fold) between these mutants. Because of this, a statistical analysis of our prior published MS findings is no longer needed.

5) We do not know why Esc2 has evolved to facilitate Mms21’s function by recruiting Ubc9. It is important to emphasize here that Esc2 partially contributes to Mms21’s function. This is supported by the overall milder defects of *esc2-D430R* than *mms21-CH* in cell growth, and DNA damage sensitivity and accumulation of GCRs, which correlate with the modestly higher sumoylation levels of Mms21-preferred targets such as MCM in the *esc2-D430R* mutant than in *mms21-CH* mutant. However, the functional relevance of MCM sumoylation is a subject of future study.

**Responses to Reviewer 2 comments:**

1) Compelling evidence describing the Esc2(Rad60)-Ubc9 binding has been reported from the structural study in *S. pombe*. Here, we confirmed that this interaction is conserved in *S. cerevisiae*. We have attempted co-IP experiments to detect the binding between endogenously expressed Esc2 and Ubc9, but have so far been unable to detect this interaction. This unpublished observation, combined with the two-hybrid over-expression study by Sollier *et al*, and the interaction between *S. pombe* Rad60 and Ubc9, which was also detected using over-expressed proteins, collectively suggest that this interaction may have a relatively modest affinity. As is often the case, the ability to detect protein-protein interactions of modest affinity via a co-IP experiment depends on the levels of proteins in the cell lysate and the non-equilibrium washing conditions used in such experiments. It is important to emphasize here that a modest affinity does not exclude the possibility that they do interact in the cell, which is “molecularly crowded”. Moreover, genetic and proteomic evidence presented in this study and the related studies of Rad60 in *S. pombe* validate the existence of Esc2-Ubc9 interaction. The observation that a single point mutation of a conserved residue in Esc2/Rad60 affects Ubc9 binding and causes a variety of *in vivo* phenotypes demonstrates the specificity of this interaction and its functions *in vivo*.
2) We appreciate the reviewer’s effort in detailing the similarity of the findings in *S. pombe* and our findings here in *S. cerevisiae*. A more explicit discussion of these results and the proposed model are included in the introduction section. Despite some differences in the severity of phenotypes that could be due to the different model organisms used, the overall mechanism is conserved. We have also included the MMS sensitivity of various *esc2* and *mms21* mutants (new Figure 4B), showing that *esc2-D430R*, unlike *esc2Δ* or *mms21-CH*, is not sensitive to MMS and does not further elevate the sensitivity of the *mms21-CH* mutant. Combined with the GCR phenotype and genetic interaction with *siz1Δ siz2Δ* mutant, *esc2-D430R* has a less severe phenotype than *mms21-CH*.
3) We have included a new MS result to compare sumoylation levels in the *esc2-D430R* and *mms21-CH* mutants (new Figure 7). This new result provides further support for their similar effects on essentially all sumoylated proteins detected in these mutants. The modestly stronger reduction of sumoylation levels of MCM by *mms21-CH* than *esc2-D430R* may contribute to its observed stronger phenotypes in genome maintenance, but the investigation into the function of MCM sumoylation is beyond the scope of this study.
4) The use of a Mms21-Ubc9 fusion protein would cause an unintended caveat, as Mms21 can no longer dissociate from Ubc9, which obviously occurs in the WT cell. While this maneuver could artificially enhance sumoylation of certain Mms21-targets in the *esc2-D430R* mutant, we feel that the new result in Figure 7 has already provided compelling evidence for the role of Esc2-Ubc9 in regulating Mms21 substrate targeting in the cell.

Minor points:

1) Line 111-113: We agree that the study of MCM sumoylation in suppressing GCR is outside the scope of this study. Thus, this discussion has been removed from the introduction and is only briefly commented on at the end of the discussion section.
2) Line 151-152, corrected.
3) Line 207, corrected.
4) Line 383-384, corrected.
5) Line 396, corrected.
